# Supplementary material for: Olfactory marker protein (OMP) regulates formation and refinement of the olfactory glomerular map
Source: Nat Commun. 2018 Nov 29;9:5073. doi: 10.1038/s41467-018-07544-9 (PMC6265328; doi:10.1038/s41467-018-07544-9)
Supplement: Supplementary file 1 — Supplemental Information [file 41467_2018_7544_MOESM1_ESM.pdf]

# Olfactory marker protein (OMP) regulates formation and refinement of the olfactory glomerular map

Albeanu et al.

**Supplementary Table 1**

**List of 99 odors for wide-field experiments, with CAS number for monomolecular odorants**

1) Peppermint oil
2) Propyl tiglate **61692-83-9**
3) Valeraldehyde **110-62-3**
4) Thiazole **288-47-1**
5) 2-acetylthiophenone **88-15-3**
6) 2-methoxy pyrazine **3149-28-8**
7) ethyl octanoate **106-32-1**
8) 4 allyl anisole **140-67-0**
9) 2,4 decadienal **25152-84-5**
10) 2,3 pentane dione **600-14-6**
11) Ethyl hexanoate **123-66-0**
12) 3’4’ dimethoxy acetophenone **1131-62-0**
13) Camphor **464-40-3**
14) Pyrolidine **52235-18-4**
15) Pyridine **85404-20-2**
16) hexanoic acid **53896-26-7**
17) heptanoic acid **111-14-8**
18) Allyl butyrate **2051-78-7**
19) Furfuryl butyrate **623-21-2**
20) ethyl valerate **539-82-2**
21) propyl butyrate **105-66-8**
22) 2,3-diethyl pyrazine **15707-24-1**
23) Hexanal **66-25-1**
24) Piperidine **110-89-4**
25) ethyl heptanoate **106-30-9**
26) Heptanal **111-71-7**
27) allyl tiglate **7493-71-2**
28) octanal **124-13-0**
29) Isoamylamine **541-23-1**
30) ethyl tiglate **5837-78-5**
31) benzyl trans 2-methyl 2-butenoate **37526-88-8**
32) Isoamylacetate **29732-50-1**
33) 2-heptanone **110-43-0**
34) methyl tiglate **6622-76-0**
35) Verbenone **80-57-9**
36) ethyl 3-mercapto-propionate **5466-06-8**
37) cedarwood oil
38) Nonanal **124-19-6**
39) 4-heptanone **123-19-3**
40) Γ terpinene **99-85-4**
41) 5-hydroxyethyl 4-methyl thiazole **137-32-6**
42) ethyl propionate **105-37-3**
43) Acetal **105-57-7**
44) 2-butenol **78-92-2**
45) ethyl butyrate **105-54-4**
46) 1-pentanol **71-41-0**
47) carvyl acetate **97-42-7**
48) Acetophenone **98-86-2**
49) Cyclohexylacetate **622-45-7**

50) 4-isopropyl benzaldehyde **122-03-2**
51) methyl pyruvate **600-22-6**
52) Eugenol **97-53-0**
53) ethyl-benzyl-acetate **94-02-0**
54) propyl acetate **109-60-4**
55) Citronellal **106-23-0**
56) 1-propanethiol **6898-84-6**
57) Isobutylamine **78-81-9**
58) DBE basic ester **106-65-0**
59) Geraniol **106-24-1**
60) Cineole **8024-53-1**
61) 2-hexanone **591-78-6**
62) furfuryl disulfide **4437-20-1**
63) isobutyl propionate **540-42-1**
64) hexanoic acid **53896-26-7**
65) 1,3- dimethoxy-benzene **28000-65-9**
66) octanoic acid **764-71-6**
67) diethyl maleate **141-05-9**
68) valeric acid **70268-41-6**
69) p-anis aldehyde **123-11-5**
70) Dyhidrocarvone **7764-50-3**
71) Decanolactone **706-14-9**
72) Fenchone **18492-37-0**
73) citral cis+trans **5392-40-5**
74) pentyl acetate **628-63-7**
75) butyl formate **592-84-7**
76) Undecane **1120-21-4**
77) butyl propionate **590-01-2**
78) allyl cyclohexane- propionate **2705-87-5** 79) butyl sulfide **544-40-1**
80) Nutmeg oil
81) Decyl alcohol **112-30-1**
82) Lemon oil
83) coffee
84) Anise oil
85) Trimethyl thiazole **13623-11-5**
86) 2-secbutyl cyclohexanone **14765-30-1**
87) Dodecyl acetate **70808-58-1**
88) 3-acetyl 2,5 dimethyl furan **10599-70-9**
89) Ethyl 3-hydroxy butyrate **5405-41-4**
90) 1,4 dimethoxy benzene **150-78-7**
91) Acetovanillone **498-02-2**
92) Indole **120-72-9**
93) 4-heptanone **123-19-3**
94) Methyl butyrate **623-42-7**
95) Nonanoic acid **112-05-0**
96) Phenyl ethyl acetate **103-45-7**
97) Hanoki oil
98) Isopropyl tiglate (1/8 nominal dilution) **1733-25-1**
99) Ethyl valerate (1/8 nominal dilution) **539-82-2**

**List of 32 odors used in two-photon imaging experiments**

1) Mineral Oil
2) 3-ethoxy,4-hydroxy benzaldehyde
3) Ethyl tiglate
4) Ethyl acrylate
5) Acetophenone
6) Allyl Tiglate
7) Valeric Acid
8) Ethyl heptanoate
9) Ethyl valerate
10) Ethyl benzoyl acetate
11) Ethyl 3-hydroxy-butyrate
12) Butyl propionate
13) Hexanoic acid
14) Ethyl propionate
15) Ethyl butyrate
16) Methyl tiglate
17) 2-ethyl burtyric acid
18) Ethyl 2-mercaptopropionate
19) Propyl acetate
20) Dimethyl phenol
21) Allyl butyrate
22) Ethyl-2-methyl butyrate
23) Heptanoic acid
24) Acetal
25) Octanoic acid
26) Isoamyl acetate
27) 2-hexanone
28) Octanal
29) Trans-2-hexanal
30) Furfuryl propionate
31) Methyl 2-pyrroll ketone 32) Furfuryl hexanoate

**List of 16 odors used in OMP-GCaMP3 imaging experiments**

1) Ethyl tiglate
2) Ethyl valerate
3) Valeric acid
4) Allyl butyrate
5) Isoamylamine
6) 2-Methoxypyrazine
7) Isugenol
8) Methyl tiglate
9) Valeraldehyde
10) Isoamyl acetate
11) Heptanal
12) mineral oil
13) Ethyl propionate
14) Propyl acetate
15) 2-Heptanone
16) Acetophenone

## Supplementary Figure 1

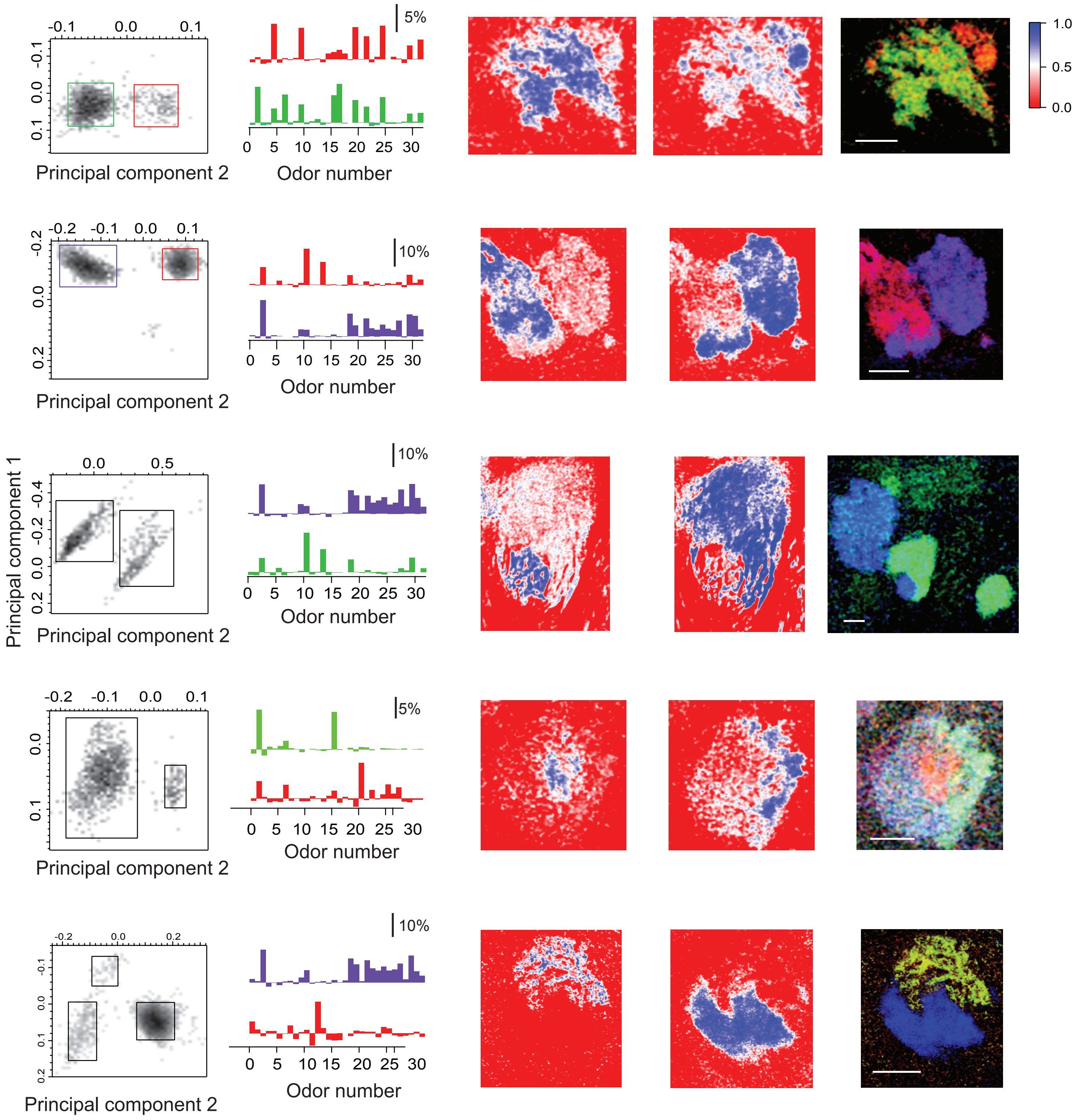


**Supplementary Figure 1. Examples of functional glomerular heterogeneity in OMP$^{-/-}$ mice.**
(*Left*) Projection of all pixels in each of the example fields of view on two principal components (PC1 vs. PC2) coordinates. PCA was performed on the odor response spectra of each pixel. (*Center*) Average odor response spectra of the functional clusters identified by PCA. (*Right*) Correlograms showing the spatial distribution of the pixel clusters identified via PCA within the example glomeruli. These were obtained by correlating the average odor response vectors of the identified clusters to the odor responses of individual pixels in each field of view. Note that each functional cluster corresponds to a spatially contiguous area, filling either a spatial microdomain within a larger heterogeneous glomerulus, or occupying the whole anatomical glomerulus. RGB color scheme overlays the correlograms corresponding to the sub-glomerular clusters. Color scale units are correlation coefficient. Scale bar, 30 μm.

## Supplementary Figure 2

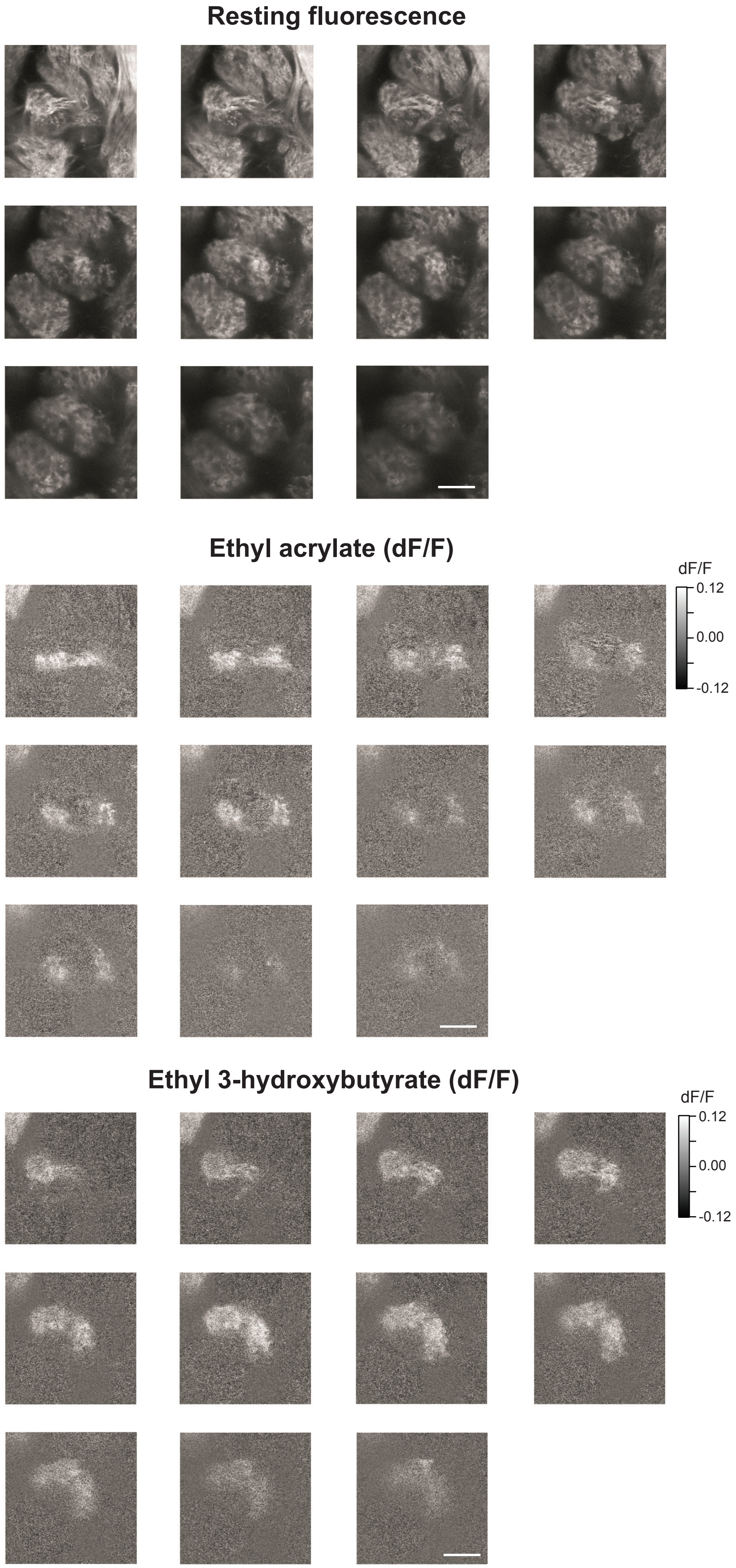


**Supplementary Figure 2. Z-stack through a functionally heterogeneous glomerulus.**
(Top) Resting fluorescence of OMP-spH labeled glomeruli sampled 8 μm apart along the z-axis. Note the different resting fluorescence intensity in different regions of the glomerulus. (Center) dF/F ratio images in response to ethyl acrylate delivery indicate two functional microdomains across different z-planes throughout the glomerulus. (Bottom) dF/F responses to ethyl 3-hydroxybutyrate (E3HB). A distinct microdomain from the one shown above (Center) was activated by E3HB. Scale bar, 40 μm

## Supplementary Figure 3

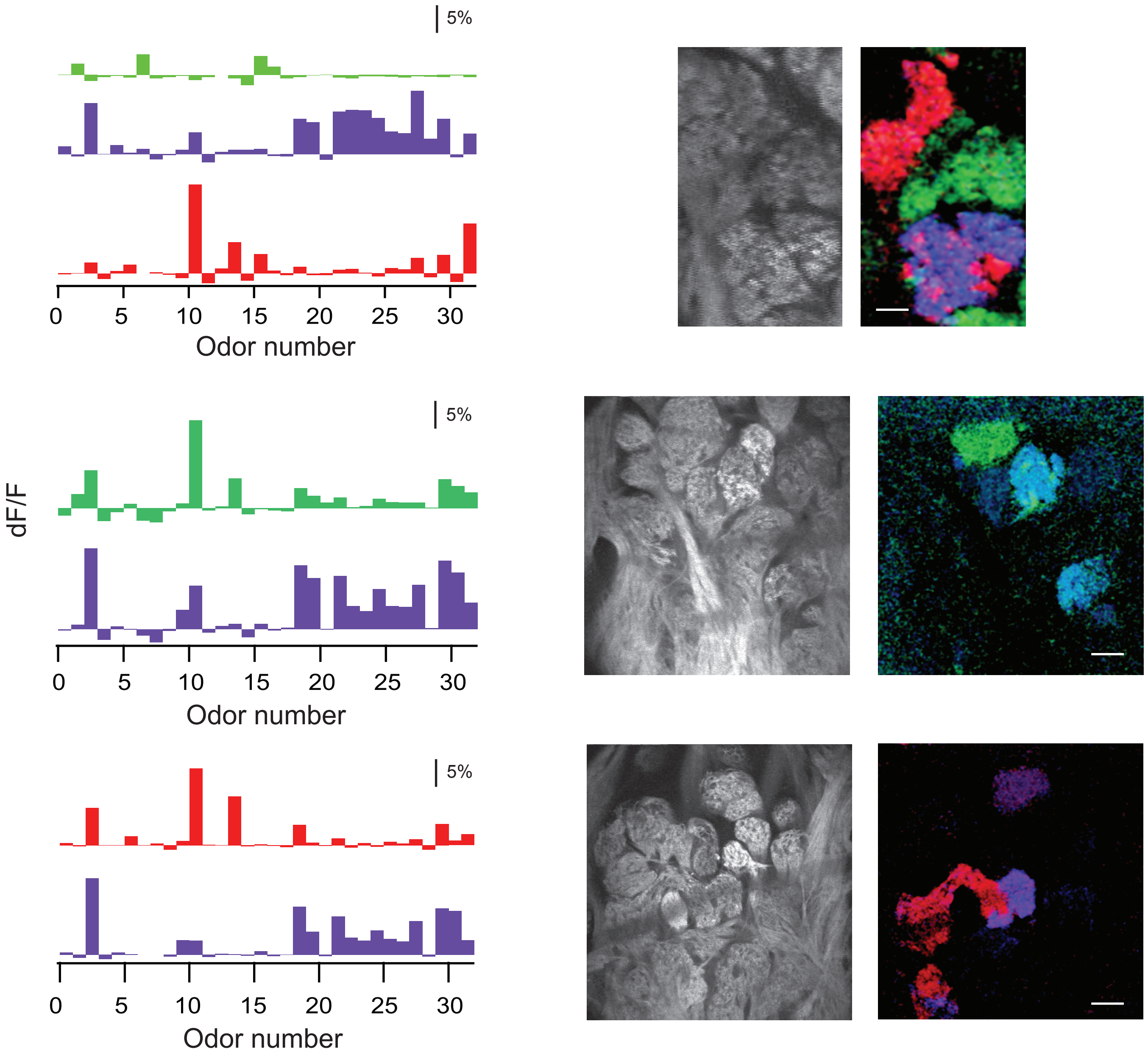


**Supplementary Figure 3. Local functional glomerular duplications in OMP$^{-/-}$ mice.**
Three example fields of view revealing functionally heterogeneous glomeruli and local duplicates. (*Left*) Example average odor response spectra of PCA identified functional clusters corresponding to glomerular microdomains. (*Right*) Resting fluorescence and overlay of correlograms. Note the presence of nearby functionally homogeneous and heterogeneous glomeruli. For example, in the top panel are depicted five anatomically identifiable glomeruli. The correlograms (*Right*) indicate that red coded microdomains are shared between two close-by anatomical glomeruli. Blue and red microdomains mix within one anatomical glomerulus. The green clusters are present as two homogeneous nearby glomeruli. Scale bar, *top* 25 µm, *middle* 60 µm, *bottom* 60 µm.

## Supplementary Figure 4

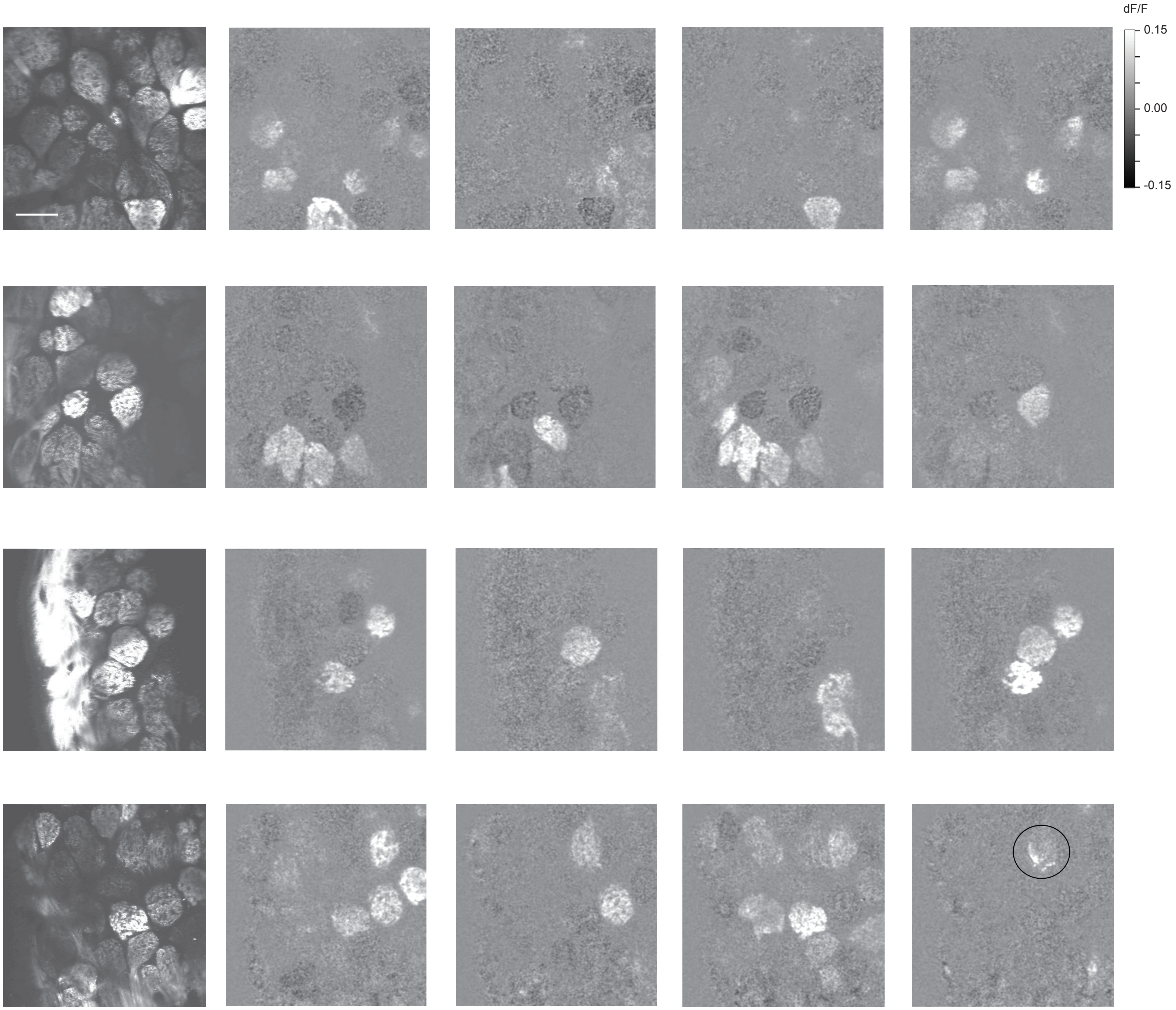


**Supplementary Figure 4. Examples of glomerular responses in OMP$^{+/-}$ mice.**
Four example fields of view from three olfactory bulb hemispheres. (*Left*) Resting fluorescence of spH labeled glomeruli in OMP$^{+/-}$ mice. (*Right*) Example glomerular odor responses (dF/F) to a panel of 32 stimuli from the fields of view shown on the left. Circle marks putative heterogeneous glomerulus. Scale bar, 100 µm.

## Supplementary Figure 5

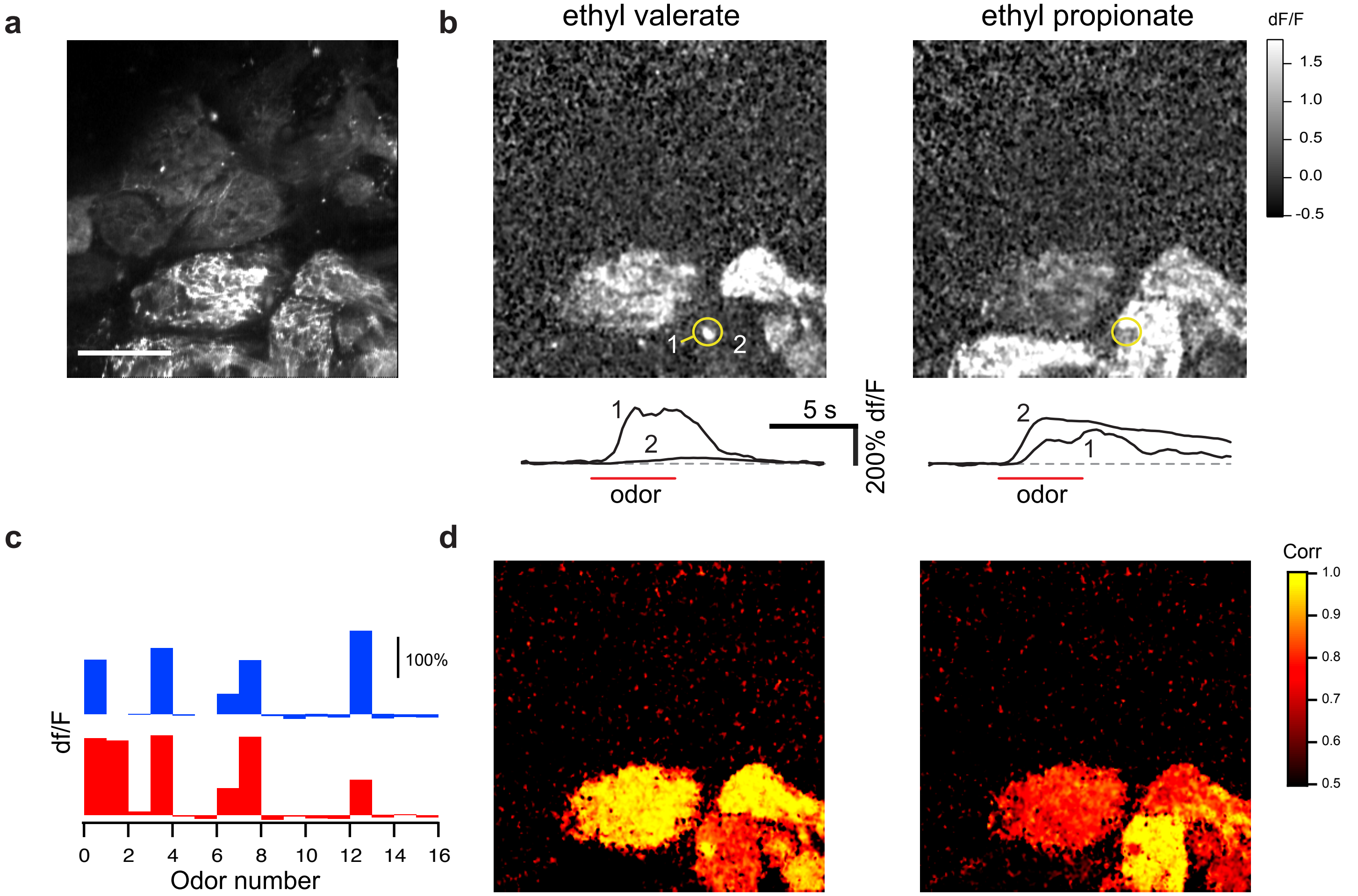


**Supplementary Figure 5. Examples of functional glomerular heterogeneity in OMP$^{+/+}$ mice.**
**a.** Resting fluorescence of example field of view. Scale bar 75 µm.
**b.** Example odor responses (GCaMP3) in the field of view shown in **a.** to ethyl valerate and ethyl propionate (1% nominal dilution).
**c.** Odor response spectra of two spatially distinct microdomains within an example glomerulus.
**d.** Single pixel correlograms of all pixels in the field of view to the odor response spectra shown in **c.** Color scale units are correlation coefficient
