# Supplementary material for: Olfactory marker protein (OMP) regulates formation and refinement of the olfactory glomerular map
Source: Nat Commun. 2018 Nov 29;9:5073. doi: 10.1038/s41467-018-07544-9 (PMC6265328; doi:10.1038/s41467-018-07544-9)
Supplement: Supplementary file 10 — Description of Additional Supplementary Files [file 41467_2018_7544_MOESM10_ESM.pdf]

Description of Additional Supplementary Files

Supplementary Movie Legends

Supplementary Movies 1 & 2.
Z-stacks of resting fluorescence through two example functionally heterogeneous glomeruli.

Supplementary Movies 3 & 4.
Example dF/F responses to 'fresh air' and different odorants in the panel within one optical plane chosen within the 2 glomeruli shown in Movies 1 & 2.

Supplementary Movies 5,6 & 7.
Example functionally heterogeneous glomerulus. Z-stacks of resting fluorescence and dF/F responses to 2 odorants in the panel (ethyl acrylate and ethyl 3-hydroxybutryate) across 38 optical planes sampled 2µm apart along the z-axis with respect to the bulb surface.
